# On the Role of Dendritic Cells Versus Other Cells in Inducing Protective CD8+ T Cell Responses

**DOI:** 10.3389/fimmu.2014.00030

**Published:** 2014-02-10

**Authors:** Rolf Martin Zinkernagel

**Affiliations:** ^1^Institute of Experimental Immunology, University Hospital of Zurich, Zurich, Switzerland

**Keywords:** crosspresentation, fibroblast APCs, tumor immunity, tolerance, lymphnode

Dendritic cells (and/or macrophages) are key transporters of antigen from extralymphatic tissue to secondary lymphatic organs. The phagocytized antigen is presented via MHC class II but not via class I, except for infections by intracellular viruses, bacteria, etc. (1–4).

Class II-negative cells (e.g., fibroblasts) that get drained to secondary lymphatic organs (including spleen) induce MHC class I restricted CD8 T cells’ cell responses as efficiently as dendritic cells (5–7).

So called crosspresentation is at least 10^5^ times less efficient than direct presentation and therefore is practically not achievable under physiological conditions (5–8).

If antigen accumulates in the endoplasmic (ER) reticulum because of transport problems, crosspresentation on to MHC class I can be demonstrated. This requires gigantic amounts of antigen accumulation in the ER, but this process has so far been difficult to quantitate in comparison to direct presentation (9).

Positive demonstration of crosspresentation in experiments is sometimes based on use of excessive amounts of protein antigen (e.g., OVA) and/or the use of unphysiological (i.e., much too sensitive) detection method, e.g., using very high frequencies of transgenic T cells (e.g., OVA-specific tgCD8^+^ T cells). In some experiments, virus inactivation is not controlled properly, permitting abortive (but not virus productive) infections that seemingly suggest crosspresentation instead of direct presentation [e.g., Ref. (8)].

An insulin-producing allogeneic cell graft strictly transplanted under the kidney capsule is accepted for more than >200 days by the host, but is promptly rejected if at the time of transplantation, or a few days later, the same cells are also given i.p. or i.v. (10) Once accepted, the allogeneic strictly peripheral cell graft is highly resistant to rejection by a transplanted corresponding allogeneic skin graft (or dendritic cells). This skin graft is rejected in a primary fashion, signaling absence of direct or indirect priming by the original allogeneic cell graft indicating absence of priming by the original cell graft. This prompt skin rejection does not cause rejection of the insulin-producing cell graft (10).

A strictly extralymphatic (7) tumor expressing a very strong and defined viral antigen (similar to insulin-producing self-beta-cells or allogeneic islet cells (10–12) can grow successfully to become lethal tumors. This depends on the condition that at the time of syngeneic tumor cell transplantation no (or too few) tumor cells escape/or drain to secondary lymphatic organs (7). This potentially early direct immunization is distinct from the late process of metastasis to secondary lymphatic organs that very often represent immune escape of tumor cells (e.g., MHC mutants, mutations of the T cell epitope, barrier formation by fibrin, coagulation, etc.)

## Discussion

DC transport antigen best to secondary lymphatic organs but only in an MHC class II associated fashion except of course if the DC is productively or abortively infected. The localization in or strictly outside of secondary lymphatic organs determines if and whether a CD8^+^ T cell immune response is induced or not. Crosspresentation of antigen to MHC class I by DC or macrophages is an experimental artifact due to overdosage or uncontrolled new cell internal synthesis. Pure crosspresentation is so inefficient, that it is largely impractical for application and therapeutic use against solid peripheral tumors.
